# Expectant mothers' not fathers' mind‐mindedness predicts infant, mother, and father conversational turns at 7 months

**DOI:** 10.1111/infa.12498

**Published:** 2022-08-26

**Authors:** Sarah Foley, Claire Hughes, Elian Fink

**Affiliations:** ^1^ Moray House School of Education and Sport University of Edinburgh Edinburgh UK; ^2^ Centre for Family Research University of Cambridge Cambridge UK; ^3^ School of Psychology University of Sussex Brighton UK

## Abstract

Parental mind‐mindedness (MM), defined as the propensity to view one's child as an agent with thoughts, feelings, and desires, is associated with positive child outcomes (McMahon & Bernier, 2017) and can be assessed in expectant parents by using five‐minute speech samples (Magaña et al., 1986). Individual differences in MM appear stable across the transition to parenthood (Foley et al., in press), offering an exciting intervention opportunity, as expectant mothers' thoughts and feelings about their unborn infants are associated with the quality of mother‐infant interactions. To assess prenatal MM as a predictor of parent‐infant conversation at 7 months, we followed 93 low‐risk British heterosexual couples across the transition to parenthood. Mothers' and fathers' MM was measured both in the third trimester of pregnancy and at 4 months. Wearable LENA devices were used to gather detailed measures of mother‐, father‐, and infant‐initiated conversations at 7 months. Prenatal MM in both parents was associated with more frequent infant‐initiated conversations at 7 months, while prenatal maternal (not paternal) MM was also associated with more mother‐ and father‐initiated conversations. While longitudinal research with more diverse samples is needed, these findings highlight the importance of parental mentalizing in the prenatal period for early family interactions.

## 1 INTRODUCTION

Pregnancy offers a window of opportunity to foster optimal child physical, social, and cognitive outcomes (Darling et al., 2020). Existing successful prevention and intervention programs have focused on promoting healthy lifestyles (e.g., nutrition, health‐promoting behaviors, breastfeeding intentions) and improving maternal perinatal mental health (O’Connor et al., 2019). Changing how expectant parents think and feel about their unborn infant may also prove fruitful in fostering sensitive and warm parent‐infant interactions (Foley & Hughes, 2018), which are known to support positive child outcomes (Mills‐Koonce et al., 2015).

Supporting this view, a study of expectant mothers (*N* = 105) showed that use of anger and anxiety descriptors of their future child when completing the Working Model of the Child Interview (WMCI; Zeanah et al., 1986) was associated with reduced warmth and sensitivity to the infant during free‐play observations at 12 months postpartum (Guyon‐Harris, Ahlfs‐Dunn, et al., 2021). However, interviews such as the WMCI are resource‐intensive and so constrain efforts to translate these research findings to clinical practice and public health policy. Furthermore, with a few exceptions (e.g., Lucassen et al., 2015), expectant fathers have been overlooked within this field of research. Addressing these gaps, the current study tested whether an easy‐to‐administer speech sample paradigm (Magaña et al., 1986) to assess expectant mothers' and fathers' representations of their unborn infants showed meaningful links to parent‐child conversations, an index of positive parent‐infant interactions that is associated with children's language development (Jaffe et al., 2001; Zimmerman et al., 2009).

A large body of research demonstrates that parents' descriptions of their children provide a sensitive index of parents' awareness of their children's emotions, cognitions, and desires, also known as mind‐mindedness (Meins et al., 2001), which is theorized to reflect the quality of dyadic relationships (Meins et al., 2014). Mind‐mindedness can be measured from parents' descriptions of their children (Meins et al., 1998) or from observations of parents with their children (Meins & Fernyhough, 2015). Individual differences in mind‐mindedness are linked to variation in attachment security in infancy (Zeegers et al., 2017), social cognition in the pre‐school years (Devine & Hughes, 2018) and other cognitive outcomes, including executive function and general cognitive ability (Aldrich et al., 2021). Despite this research, a review of 20 years of research on parental mind‐mindedness identified three noteworthy gaps (McMahon & Bernier, 2017).

First, studies of associations between mind‐mindedness and quality of caregiving have largely relied upon brief observations, typically to assess maternal sensitivity (Zeegers et al., 2017), which are often conducted in lab settings and so may compromise their ecological validity (Holden et al., 2014). In the current study, we countered these problems by gathering day‐long naturalistic recordings of parent‐child interactions, using the Language Environment Analysis system (LENA, 2018), which comprises a wearable audio recording device and software for automated analysis. While early research using LENA focused on the number of words heard by the child, subsequent work demonstrates that, over and above overall adult word counts, the frequency of conversational turns matters for children's later language (Zimmerman et al., 2009). Furthermore, this dyadic measure of parent‐child conversation is also indicative of the quality of emerging parent‐child relationships. For example, coordinated parent‐child turn‐taking at 4 months of age predicts attachment security at 12 months of age (Jaffe et al., 2001), while frequency of conversational turn‐taking between parent and child at 18 months of age predicts quality of parent‐child interaction during free‐play at 30 months of age (Gomez & Strasser, 2021). Thus, in the current study LENA measures of parent‐child conversations were our outcomes of interest. We hypothesized that parents who view their infants as mental beings with independent thoughts and feelings will: (a) initiate a greater number of conversations with their infants (Fernyhough, 2008); and, as a result of this increased parental talk (b) have infants who are also more likely to initiate conversations.

A second gap in the mind‐mindedness literature concerns the paucity of studies that include the prenatal period. One pioneering study of 25 expectant couples (68% first‐time parents), showed that most struggled to describe their unborn infant, but those who could say something were, at 6 months postpartum, more likely to make appropriate mind‐related comments toward their infants (Arnott & Meins, 2008). Prenatal mentalistic attributes were coded simply as either present or absent; fathers (but not mothers) who could provide a mind‐minded description showed more frequent appropriate mind‐related comments at 6 months. Expanding this work, recent research (Foley et al., in press) involving 198 first‐time mothers and fathers followed across four time points from late pregnancy to 24 months postpartum showed an increase over time in mean levels of mind‐mindedness, coupled with modest but significant temporal stability for individual differences. Importantly, for both expectant mothers and fathers, individual differences in prenatal mind‐mindedness were unrelated to parents' education, perceived social standing, indices of poor mental health, anticipated involvement in childcare, or knowledge of infant sex. This evidence of discriminant validity adds further weight to the hypothesis that the tendency to think of one's future infant as a sentient being does not reflect differences in parents' state of mind (Larkin et al., 2021). Furthermore, the similarity between expectant mothers and fathers demonstrates that a physical link is not a prerequisite for a psychological connection. Given the challenge of teasing apart child and parental contributions to relationship quality, assessing parental mind‐mindedness in the prenatal period may help provide a clearer picture of the likely role of parental thoughts and feelings about the infant in shaping interactions. However, direct evidence for a unique association between prenatal mind‐mindedness and parent‐infant interaction quality remains lacking. To address this gap, the current study included speech‐sample‐based ratings of mind‐mindedness in the third trimester of pregnancy and at 4 months postpartum. We hypothesized that, by indexing parents' very early propensity to adopt a mentalistic stance toward their infant, the first (prenatal) measure of mind‐mindedness would be positively associated with parent‐infant conversations at 7‐month, even when the postnatal measure was included in the model.

The third gap in the mind‐mindedness literature concerns the neglect of fathers, limiting our understanding of both the applicability of maternal findings to fathers and within‐couple effects. A review of three decades of parenting research demonstrated that, alongside parent and child characteristics, contextual factors influence parents' cognitions and behaviors (Taraban & Shaw, 2018). For example, prior research with the current sample highlighted poor couple relationship quality mediated the association between poor parental well‐being during pregnancy and toddler internalizing problems at 24 months (Hughes et al., 2020). With this in mind, we aim to examine whether, alongside within‐person associations, measures of parental mind‐mindedness are associated with *partners*' initiations of conversations with their infant. That is, whether early mothers' mind‐mindedness will impact fathers' initiations of conversations with their infant or vice versa. This question has clear practical implications, as evidence for partner influence would highlight the importance of including both parents in interventions.

In sum, in the current study we assessed whether individual differences in mothers' and fathers' prenatal and postnatal mind‐mindedness were associated with variation in the frequency of conversation as initiated by themselves, their partner, and their infant at 7 months of age. We hypothesized that expectant parents' ability to view their unborn infant as a sentient being would be associated with greater frequencies of both self‐ and infant‐initiated conversations at 7 months postpartum. We adopted an exploratory approach in terms of whether the associations would be similar or distinct for mothers versus fathers. Note that we chose to focus on 7 months of age to capture very early infant talk, which emerges after approximately 6 months, as indicated by a shift from unilateral to more symmetrical communication and mutual engagement (Hsu & Fogel, 2003) and a dramatic reduction in infants vocalizing over their parents talk between 5 and 9 months (Hilbrink et al., 2015). Our dual focus on mothers and fathers also enabled us to compare maternal versus paternal influences on family talk.

## 2 METHODS

### 2.1 Sample

We recruited 93 expectant couples attending antenatal clinics, ultrasound scans, and parenting fairs in the East of England. Most participants (*n* = 71) were recruited for the New Fathers and Mothers Study (Hughes et al., 2018), while an additional 17 were recruited from the maternity unit of a local hospital, three couples were recruited from a shared database of families from the Rosie Hospital, Cambridge, and a further two couples were recruited via word of mouth. To be eligible, participants had to: (i) be first‐time parents, (ii) expecting delivery of a healthy singleton baby, (iii) planning to speak English as a primary language with their child, and (iv) have no history of severe mental illness (e.g., psychosis) or substance misuse. At the birth of their baby, mothers were, on average, 31.99 years old, *SD* = 4.42 years, range: 20–45 years, and fathers were, on average, 33.54 years old, *SD* = 5.96 years, range: 21–50 years. Most of the sample were highly educated (83% of mothers and 74% of fathers had an undergraduate or higher degree), a minority of parents were from ethnic minority backgrounds (9% of mothers and 8% of fathers). All infants (52% male) were born healthy, as confirmed by midwife report, and, according to parent report on the Ages and Stages Questionnaire, showed typical development at 4 months of age (*M* = 4.16 months, *SD* = 0.50) when the postnatal speech sample was collected and at 7 months of age (*M* = 6.94 months, *SD* = 1.09) when the LENA measures were collected (for more information see Fink et al., 2019). Five‐minute speech samples were completed by 92 mothers and 89 fathers prior to the birth of their child and by 88 mothers and 86 fathers at 4 months postpartum. We adopted a full information approach to data analysis using the complete sample of 93 families.

### 2.2 Procedure and measures

The present study was conducted according to guidelines laid down in the Declaration of Helsinki, with written informed consent obtained from parents before being interviewed in the third trimester, at 4 months, and also for each child before any assessment or data collection involving each infant at 7 months. All procedures involving human subjects in this study were approved by the National Health Service (NHS UK) Research Ethics Committee (London Bloomsbury, Protocol ref: 14/LO/1113). Parents also completed online questionnaires about their well‐being and family background. At 7 months of age, LENA devices were posted to families, along with detailed instructions for use. Families were asked to record a typical day in the life of their infant when both mother and father were caregiving.

**Mind‐Mindedness.** At both time points parents provided a five‐minute speech sample describing their (future) infant and their (future) relationship with their child (Magaña et al., 1986). These speech samples were audio‐recorded, transcribed verbatim, anonymized, and coded for mind‐mindedness (Meins & Fernyhough, 2015). The study team had prior experience coding mind‐mindedness from parents' descriptions (Hughes et al., 2017) and the coders were not involved in the gathering or processing of data collected via Language Environment Analysis (LENA, 2018). Coding the representational measure of mind‐mindedness from the transcripts required identification of all attributes that referred to the child and subsequently these attributes were coded as mental (e.g., including cognitions, emotions, desires, e.g., during pregnancy, “He'll be a happy little chap”, or at 4 months, “She's stubborn”) or non‐mental (e.g., including general descriptors or physical and behavioral attributes, e.g., during pregnancy, “it is going to be hopefully perfectly healthy”, or at 4 months, “She's a bundle of energy”). Frequencies of mental and non‐mental child attributes were used to construct proportional scores, to control for variation in parental fluency or verbosity. At both time points, inter‐rater reliability based on 20% of the speech samples was excellent for mental (prenatal ICC = 0.81, 4 months ICC = 0.75) and non‐mental attributes (prenatal ICC = 0.91, 4‐month ICC = 0.84). The reliability set was divided in half to check for coder drift during the coding process.

**Family Member‐Initiated Conversational Turns.** To collect information on family conversational turns across the day, the Language Environment Analysis (LENA) device (LENA, 2018), which comprises a small audio recorder housed in a bespoke vest, was worn by the infant for a full day (maximum 16 h). The average duration of recordings was 15.42 h (*SD* = 1.47 h; range = 5.50–16.00 h). LENA has been shown to be reliable for English‐speaking families (Oetting et al., 2009; Oller et al., 2010; Xu et al., 2009), and a manual reliability check for this data (Fink et al., 2019) showed that manual conversational turn counts were similar to those extracted by the LENA software, ICC = 0.86 (95%CI = 0.67 ‐ 0.94). Conversational turns data were extracted using the LENA software (for technical reports, see Ford et al., 2009) and then processed by the Advanced Data Extractor (ADEX) software to provide information on the frequency of mother‐, father‐, and child‐initiated conversational turns throughout a single day. Conversational turns are defined by LENA as at least a one adult/child utterance followed by a reciprocated child/adult utterance with a maximum intervening interval of 5 s of silence. Only conversational turns that included a child were processed (i.e., conversations between parents were not included). An hourly rate of conversational turns was used in the analysis to account for differing lengths of audio recordings.

**Demographics.** At the prenatal time point, parents provided information about their age, education level, ethnicity, and rated their perceived social standing on a 10‐point ladder (Singh‐Manoux et al., 2003).

## 3 RESULTS

### 3.1 Preliminary analyses

First, we examined the independence of mother and father mind‐mindedness. Non‐independence of parental mind‐mindedness data would mean that analysis would need to consider the dyadic nature of the couple. As mothers and fathers comprised distinguishable dyads, we tested for independence in mind‐mindedness using bivariate correlations (Kenny et al., 2006) (Kenny et al., 2006). Non‐significant correlations between mothers and fathers both prenatally, *r*(88) = 0.14, *p* = 0.190 and postnatally, *r*(88) = 0.12, *p* = 0.270, supported the appropriateness of proceeding with the individual as the unit in our analyses.

Next, we examined associations between demographic characteristics, mind‐mindedness, and the family conversational talk variables (see Tables 1 and 2). Examining study variables by maternal education showed that parental mind‐mindedness (either mother or father; prenatal or 4‐month) was unrelated to whether mothers had a degree, *t*s < 0.81, *p* > 0.419. However, children at 7 months were more likely to initiate conversations with their parents if their mother had a tertiary degree (*n* = 69, *M* = 11.26, *SD* = 4.17), compared with children whose mother did not have a tertiary degree (*n* = 23, *M* = 8.81, *SD* = 2.68), *t* (90) = 2.63, *p* = 0.010, Cohen's *d* = 0.64. There was no difference in mother‐ or father‐initiated conversation as a function of mothers' education, *t*s < 0.1.52, *p* > 0.132. There was a significant positive association between mothers' age and mother‐initiated conversation, such that older mothers were more likely to initiate a greater number of conversations with their infants than younger mothers, *r* (86) = 0.25, *p* = 0.022. All other study variables were unrelated to mothers' age, *r*s < 0.19, *p* > 0.082.

**TABLE 1:** Descriptive statistics for study variables. (Gender differences for prenatal mind‐mindedness is not reported as not all families knew the sex of the baby prior to birth.)

		Total sample			Boys (*n* = 48)	Girls (*n* = 45)		
		*N*	*M* (SD)	Range	*M* (SD)	*M* (SD)	*t*	Cohen's *d*
Prenatal	Mother MM	92	0.26 (0.16)	0–0.73	–	–	–	–
	Father MM	89	0.27 (0.20)	0–0.73	–	–	–	–
4‐ months	Mother MM	88	0.39 (0.14)	0.08–0.69	0.38 (0.14)	0.39 (0.13)	−0.34	0.07
	Father MM	86	0.36 (0.14)	0–0.67	0.32 (0.14)	0.40 (0.14)	−2.36*	0.51
7 months	Infant age (months)	93	6.87 (1.09)	5–9	6.94 (1.04)	6.80 (1.14)	0.61	0.13
	Infant‐initiated conversation	93	10.68 (3.98)	1.6–22.1	10.94 (4.37)	10.40 (3.5)	0.66	0.14
	Mother‐initiated conversation	93	9.98 (3.38)	2.3–20.4	9.94 (3.70)	10.01 (3.04)	−0.10	0.02
	Father‐initiated conversation	93	4.23 (2.40)	0.5–13.2	4.14 (2.32)	4.32 (2.50)	−0.37	0.08

*Note*: MM = mind‐mindedness.

*p < 0.05, **p < 0.01.

**TABLE 2:** Summary of bivariate correlations between study variables above the diagonal, controlling for infant age at 7 months below the diagonal. (Infant age associations with mind‐mindedness variables are not reported as age was collected at 7 months)

		1	2	3	4	5	6	7	8
Prenatal	1. Mother MM	‐	0.14	0.29**	−0.03	‐	0.23*	0.15	0.24*
	2. Father MM		‐	0.05	0.01	‐	0.20	0.19	0.22*
4 months	3. Mother MM			‐	0.12	‐	0.11	0.02	0.14
	4. Father MM				‐	‐	0.07	0.00	−0.03
7 months	5. Infant age	‐	‐	‐	‐	‐	0.10	0.04	0.08
	6. Infant‐initiated conversation	0.21^+^	0.22	0.07	0.10	‐	‐	0.60**	0.48**
	7. Mother‐initiated conversation	0.14	0.23*	−0.04	0.05	‐	0.57**	‐	0.17
	8. Father‐initiated conversation	0.22^+^	0.23*	0.13	−0.05	‐	0.36**	0.14	‐

*Note*: MM = mind‐mindedness.

^+^ *p* < 0.10, *p < 0.05, **p < 0.01.

For fathers, degree status was unrelated to mind‐mindedness, *t*s < 1.29, *p* > 0.204. However, mothers whose partners had a tertiary degree had higher postnatal mind‐mindedness scores (*n* = 55, *M* = 0.42, *SD* = 0.14), compared with mothers whose partners did not have a tertiary degree (*n* = 31, *M* = 0.34, *SD* = 0.13), *t* (84) = 2.43, *p* = 0.017, Cohen's *d* = 0.59. Fathers' degree status was unrelated to any of the family conversational talk variables, *t*s < 0.1.25, *p* > 0.215; fathers' age was also unrelated to the study variables, *r*s < 0.14, *p* > 0.209.

Infant age on the day of the LENA recordings was unrelated to family conversation scores. While fathers with daughters appeared more mind‐minded than fathers with sons, infant sex and age were largely unrelated to study variables (see Tables 1 and 2).

### 3.2 Family member‐initiated conversations

On average, 7‐month‐old infants initiated almost 11 conversations per hour, mothers initiated almost 10 conversations per hour, and fathers initiated 4 conversation per hour (see Table 1). The frequency of conversations initiated by infants, mothers, and fathers were all significantly correlated. Moreover, there was considerable variability in conversation length across families: while 50% of families had a maximum of 11 turns within a single conversation, 10% of families had a maximum conversational turn length of between 29 and 43 turns.

A repeated‐measures ANCOVA, with family talk as the repeated measure (i.e., infant, mother‐, and father‐initiated conversation scores) and infant age as the covariate was conducted to examine mean level differences in initiated conversations across family members. The ANCOVA showed an overall difference in the frequency of conversations across family members, *F* (2, 182) = 3.96, *p* = 0.021, η^2^_p_ = 0.042. Post hoc pairwise contrasts showed that father initiated significantly fewer conversations than either mothers or infants, *p*s < 0.001, but there was no significant difference in the frequency of infant‐ and mother‐initiated conversations, *p* = 0.135.

### 3.3 Parental mind‐mindedness and family member‐initiated conversations

Given the independence of mother and father mind‐mindedness scores (Table 2), two separate multivariate regression models were constructed in M*plus* (Muthén & Muthén, 1998–2017) to examine the role of parental mind‐mindedness for later initiated conversations. An MLR estimator was used to account for missing data. In both models, infant‐, mother‐, and father‐initiated conversations were included as the outcome and were permitted to be correlated. Both models controlled for parental education level and infant age on the day the LENA recording took place.

**FIGURE 1:**
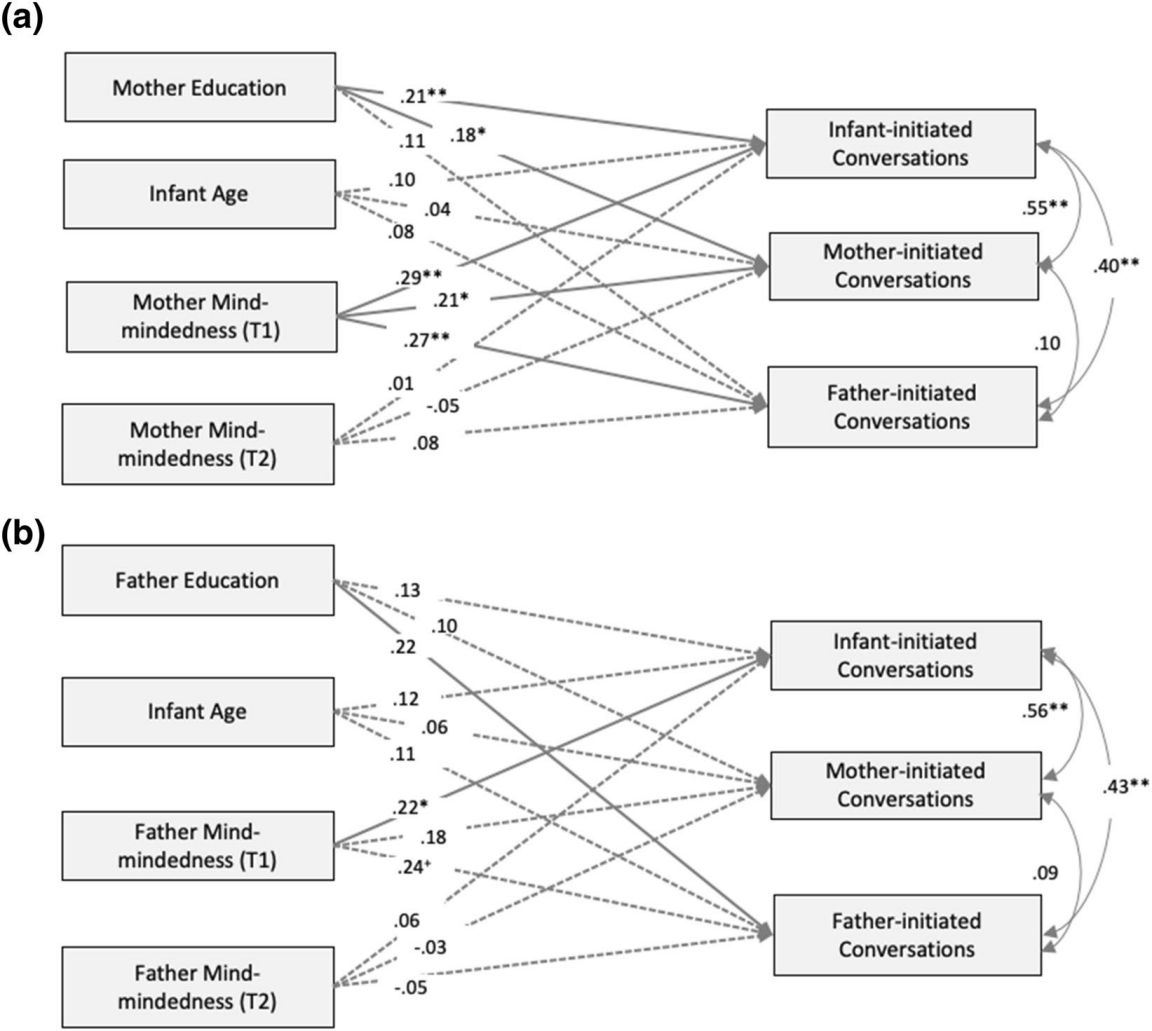
Standardized coefficients of the links between expectant mothers' (a) and fathers' (b) mind‐mindedness and infant, mother‐, and father‐initiated conversation turns. *p < 0.05, **p < 0.01

In the first model (Figure 1, Model A), expectant mothers' mind‐mindedness predicted the frequency of infant‐, mother‐, and father‐initiated conversations, controlling for mothers' education level and child age at time of LENA recording. By contrast, there was no significant prospective association between maternal mind‐mindedness at 4 months and any conversation outcome. In the second model (Figure 1, Model B), prenatal ratings of fathers' mind‐mindedness was only associated with infant‐initiated conversation while controlling for fathers' education level and child age at time of LENA recording, and like mothers, there was no significant prospective association between fathers' mind‐mindedness at 4 months and any conversational outcomes.

## 4 DISCUSSION

We followed 186 new mothers and fathers (93 couples) across the transition to parenthood and assessed the level of mind‐mindedness in their representations of their infant, during the last trimester of pregnancy, and at 4 months. At 7 months we used LENA to capture the frequency of naturally occurring conversational bouts, initiated by infant, mother, or father. Our analysis demonstrated the importance of expectant parents' mind‐mindedness for infant‐initiated talk, with expectant mothers' mind‐mindedness additionally explaining both her own and her partner's talk. Importantly, these associations remained significant even when postnatal mind‐mindedness was included in the model. Together, these findings highlight the importance of parental mentalizing in the prenatal period for early family interactions. Below, we discuss these findings and consider their implications for future research and intervention.

We believe that the current study is the first to consider prenatal mind‐mindedness as a predictor of the dynamics of parent‐infant conversation during naturalistic parent‐child interactions, which may be considered an index of parent‐infant relationship quality. Beyond postnatal measures of mind‐mindedness and age‐related changes in talk, expectant mothers' tendency to describe their unborn infants in terms of thoughts, feelings, and desires was associated with more frequent mother‐, infant‐, and father‐initiated conversational turns.

In line with the prototype hypothesis (Fraley et al., 2013), the representations that parents begin to form of their future relationship with their infant during pregnancy reflect legacies of their own earlier relationships and appear robust to early child influences and initial postnatal representations in the first year of life (Madigan et al., 2015). Thus, expectant mothers who, compared with their peers, were ahead of the curve in forming mind‐minded anticipatory working models (Heinicke et al., 1983; Zeanah & Benoit, 1995), may have also been quicker to demonstrate sensitivity to their infants' cues. Consequently, once their infants mastered making sounds, they may have elicited more responses from their primary caregivers (in this case mothers) and hence been more motivated to initiate conversations themselves (Matthews, 2020).

Interestingly, while mind‐minded expectant fathers were not themselves more likely to initiate conversations, their infants were. This suggests that these fathers perhaps made themselves available to their infants in different non‐verbal ways, for example, through play, which would not be captured by LENA. These findings are consistent with meta‐analytic results that interview and questionnaire ratings of maternal but not paternal prenatal fetal attachment are associated with observed sensitivity during the first years of life (Foley & Hughes, 2018). As the five‐minute speech sample paradigm combines the brevity of questionnaires with the detail of interview approaches, the current findings offer an opportunity for future studies to adopt a methodology that is quick to administer and yet allows researchers to gather a rich picture of the parents' developing relationship (Sher‐Censor, 2015). Indeed, the lack of within‐couple concordance in mind‐mindedness provides further support for the relationship‐specific nature of mind‐mindedness (Meins et al., 2014). Future research examining associations between mind‐mindedness, other parental mentalizing dimensions (e.g., parental reflective functioning; Fonagy et al., 1991) and general mentalizing abilities (e.g., Unknown Mother‐Infant Interaction Task; Larkin et al., 2021) and prospective links with parent‐child interaction quality, will help tease apart the relative overlap between specific representational dimensions and their unique contribution to later parent and child outcomes.

Variation in expectant mothers' mind‐mindedness was also associated with variation in fathers' conversation initiation at 7 months. Interestingly, this association could not be attributed to a general within‐couple similarity between mothers' and fathers' child‐directed talk, for two reasons. First, mothers' and fathers' child‐directed talk showed no strong association. Second, our model included the frequencies of mother‐ and infant‐initiated conversations. Future research is needed to examine whether mothers' prenatal mind‐mindedness does indeed facilitate their partner's talk and what the potential mediators of this effect are. For example, these findings are consistent with the broader mentalizing literature that suggests that adults who can tune into their own and other people's mental lives may be more adept at getting others to talk (Luyten et al., 2020). However, while mind‐minded mothers may encourage their partners to develop an interaction style that elicits infant responses; further work is needed to establish whether this encouragement is deliberate or unintentional.

While intriguing, the asymmetry between mothers and fathers should be interpreted with caution, especially given the impact of paternal mind‐mindedness on infant talk in the family. In particular, our findings are based on a demographically homogenous sample of first‐time mothers and fathers and so may not extend to more diverse samples (c.f., Casillas et al., 2020). Despite the fact there was no association between maternal and paternal mind‐mindedness, a larger more diverse sample would also allow us to examine the impact of mothers' and fathers' on family talk measures simultaneously. In addition, while LENA recordings were carried out on days when both parents were present, the primary caregivers were almost exclusively mothers, such that any contrast may reflect partner differences in overall time spent with the infant. An obvious means of distinguishing effects of gender versus caregiver role would be to recruit primary‐caregiver fathers or same‐sex parents. Equally, it would be useful to include multiple time points to gather data across an extended developmental period, as fathers may become more involved as children grow up (Cano et al., 2019). Finally, while the representational measure makes it possible to capture variability in mind‐mindedness during pregnancy and stability across the transition to parenthood, the method does not enable clarifying whether mind‐related comments appear to be appropriate or non‐attuned. There was also a short time interval between our postpartum measure of mind‐mindedness and the LENA outcomes. Thus, although mind‐mindedness appears to be stable in the early years of a child's life, further work with concurrent measures is required to clarify this association.

Despite these limitations, our findings open exciting possibilities for future research. For example, while we used the LENA measure of conversational turns, it is also possible to use LENA data as a naturalistic marker of children's language development. Recent meta‐analytic evidence suggests a modest but significant association between parental mind‐mindedness and children's expressive and receptive vocabulary, language production, and verbal IQ (Aldrich et al., 2021). Building on the current findings, future longitudinal research into associations between mind‐mindedness and naturalistic indices of family talk may shed light on the mechanisms that underpin this link between parent mind‐mindedness and children's language development.

In addition, while the majority of LENA research to date has focused on counts of adult and child words, as well as an overall tally of parent‐child conversations through the course of a day, the current study suggests that more fine‐grained data about the frequency and speaker identity for individual conversational initiation between parent and infant yield insights into family dynamics and, importantly, show meaningful links with psychological parenting constructs. These findings highlight the value of extending the use of LENA‐extracted data to consider the quality of dyadic interaction (e.g., Gomez & Strasser, 2021). In line with these nuanced findings, in our future work we plan to examine the content of both the parent‐child conversations as well as the affective quality and coherence of speech‐sample descriptions (Demers et al., 2010; Sher‐Censor et al., 2018). Research with larger and more diverse samples will also be helpful in identifying factors that amplify or attenuate associations between expectant mothers' mind‐mindedness and family talk in infancy. For example, the relationship between mother mind‐mindedness and father conversation initiation may be stronger in the context of high couple satisfaction (Fink et al., 2019) or co‐parenting quality (Quigley & Nixon, 2022).

In conclusion, the nature of expectant parents' thoughts and feelings about their infants appears to matter for the quality of later parent‐child interactions (Foley & Hughes, 2018; Guyon‐Harris, Ahlfs‐Dunn, et al., 2021). Results from postnatal mind‐mindedness interventions show promising shifts in parents' behaviors and infant attachment security (Larkin et al., 2019). Evidence from future research to test if our findings replicate with larger diverse samples and across a range of parent and child outcomes will be helpful in establishing whether antenatal healthcare professionals should broaden or shift the focus of their interventions to enhance mind‐mindedness in expectant parents.

## CONFLICT OF INTEREST

The authors declare no conflicts of interest with regard to the funding source for this study.

## Data Availability

Data are available from the UK Data Service: Hughes, C., Devine, R.T., Mesman, J., & Blair, C. (2018). *The New Fathers and Mothers Study.* Colchester, Essex: UK Data Service. 10.5255/UKDA‐SN‐853278.
